# Petri net–based model of the human DNA base excision repair pathway

**DOI:** 10.1371/journal.pone.0217913

**Published:** 2019-09-13

**Authors:** Marcin Radom, Magdalena A. Machnicka, Joanna Krwawicz, Janusz M. Bujnicki, Piotr Formanowicz

**Affiliations:** 1 Institute of Computing Science, Poznan University of Technology, Poznań, Poland; 2 Institute of Bioorganic Chemistry, Polish Academy of Sciences, Poznań, Poland; 3 Laboratory of Bioinformatics and Protein Engineering, International Institute of Molecular and Cell Biology, Warsaw, Poland; 4 Institute of Informatics, University of Warsaw, Warsaw, Poland; 5 Institute of Biochemistry and Biophysics, Polish Academy of Sciences, Warsaw, Poland; 6 Laboratory of Structural Biology, International Institute of Molecular and Cell Biology, Warsaw, Poland; 7 Institute of Molecular Biology and Biotechnology, Faculty of Biology, Adam Mickiewicz University, Poznań, Poland; Instituto Gulbenkian de Ciência - IGC, PORTUGAL

## Abstract

Cellular DNA is daily exposed to several damaging agents causing a plethora of DNA lesions. As a first aid to restore DNA integrity, several enzymes got specialized in damage recognition and lesion removal during the process called base excision repair (BER). A large number of DNA damage types and several different readers of nucleic acids lesions during BER pathway as well as two sub-pathways were considered in the definition of a model using the Petri net framework. The intuitive graphical representation in combination with precise mathematical analysis methods are the strong advantages of the Petri net-based representation of biological processes and make Petri nets a promising approach for modeling and analysis of human BER. The reported results provide new information that will aid efforts to characterize *in silico* knockouts as well as help to predict the sensitivity of the cell with inactivated repair proteins to different types of DNA damage. The results can also help in identifying the by-passing pathways that may lead to lack of pronounced phenotypes associated with mutations in some of the proteins. This knowledge is very useful when DNA damage-inducing drugs are introduced for cancer therapy, and lack of DNA repair is desirable for tumor cell death.

## Introduction

Living organisms as well as their functional parts such as organs, tissues, cells, etc. are very complex systems. These systems are composed of numerous basic building blocks connected by a dense network of interactions and mutual dependencies. A sum of such networks determines the structure and the functionality of the system. Therefore, in order to fully understand the nature of living organisms, it does not suffice to analyze properties of the building blocks, but it is also necessary to analyze the networks of interactions among them. Thus far, various computational methods for analyzing complex systems have been developed, and some of them can be applied in the analysis of biological systems [1, 2]. However, these systems have their own specificity, from which follows that in some cases it can be difficult to use methods developed earlier for other systems.

The first and necessary step in the analysis of a complex system is to build its formal model. This model can be expressed in a language of some branch of mathematics, and for instance, differential equations are often used for this purpose. Recently, Petri net theory has emerged as a promising approach for modeling and analysis of biological systems [1]. These nets have been described for the first time by C. A. Petri in the early 1960s in the context of theoretical computer science and for many years this was the main area of their applications [3, 4]. Since this time the theory of Petri nets has been intensively studied and many methods of the analysis of their properties have been developed. In the mid 1990s it was realized that Petri nets could also be used for modeling and analysis of biological systems [5, 6] and during the last two decades the possibilities and limitations of Petri net applications in this area were studied [1, 7, 8].

On the one hand, a strong advantage of Petri net-based models is their intuitive graphical representation. On the other hand, the nets can be analyzed using precise mathematical methods. Moreover, contrary to differential equations, they do not require the knowledge about precise values of some parameters of the modeled system, what can be a very important property in many cases since these values are often difficult to determine for biological phenomena.

The integrity of genetic information depends on the interplay between the DNA base modification (caused by enzymatic reactions), DNA damage (caused by cellular and environmental chemical reactions), and all DNA-binding proteins and readers of information that work together to make the whole system functional. The DNA damage repair is an exemplary complex biological system, in which a large number of elements (mostly enzymes that perform chemical reactions) cooperate with each other to protect the cell from consequences of DNA damage events. Base damage can be mutagenic and/or cytotoxic and can lead to mutagenesis or cell death, respectively, when unrepaired DNA lesions undergo replication or transcription. To avoid such consequences, correct repair of different types of DNA damage is assured by the existence of several DNA repair subsystems maintained by the cell in order to survive the DNA damage. In humans these subsystems, called pathways, include DNA damage signaling (DDS), direct reversal repair (DRR), base excision repair (BER), nucleotide excision repair (NER), mismatch repair (MMR), homologous recombination repair (HRR), nonhomologous end-joining (NHEJ) and translesion synthesis (TLS). Details concerning DNA damage and repair in several living organisms were collected and included in the public databases developed by our groups (http://repairtoire.genesilico.pl/pathways/ [9], https://www.ibb.waw.pl/pl/Sekretariat%20Naukowy/Projekty%20strukturalne/dnatraffic [10]).

The DNA repair processes have already been a subject of computational modeling. A broad range of modeling approaches gave opportunities to gain a variety of insights into DNA damage and repair. Some of these models were built based on detailed kinetic data available from experimental studies. Examples include the stochastic model of NHEJ, which uses molecule numbers, reaction rate constants and kinetic rate constants to explain the dynamic response to damage induced by different levels of gamma irradiation in human fibroblasts [11]. Computational modeling was also conducted for some sub-pathways of BER, where e.g., differential equations were used to model the removal of 8-oxoguanine (8-oxoG) *via* base excision repair initiated by human OGG1 glycosylase [12]. In this case the availability of kinetic data for both wild type enzymes and their mutant variants allowed for the prediction of the kinetics and capacity of the whole sub-pathway in the presence of some single-nucleotide polymorphisms which have been associated with cancer. Other examples of kinetic models for BER include the works of [13] and [14]. Valuable insights into DNA repair processes were also delivered by models which did not require incorporation of very detailed data as in the cases discussed above. For example, Monte Carlo simulations of radiation-induced damage and its repair were used to investigate the repair of clustered damage *via* BER and NER pathways [15, 16]. These approaches allowed to estimate the probabilities of correct repair, fractions of damages converted into double-strand breaks (DSBs) and fractions of repairs ending with mutations.

In this work, we present an extensive study of a Petri net model of the BER repair pathways, which we have built based on available literature and from biological pathway databases. In contrast to models built by others, our approach involves modeling the repair of many different types of DNA damage through the activity of a collection of DNA glycosylases initiating BER and other enzymes performing further steps of the repair. With such a comprehensive model formal analysis becomes possible using methods provided by the Petri nets theory. Here we have performed a t-invariant based analysis, involving MCT sets and t-clusters, decomposing the model into precisely identified and named functional modules. Understanding their interaction provides valuable insights into the complex DNA repair process. We have also performed an *in silico* knockout analysis as a prediction of system behavior under a collection of disturbances.

The organization of the paper is as follows. Section 2 provides an overview of Petri nets, including the analytical analysis of t-invariants. In Section 3 the results of the net analysis are presented. The paper ends with discussion and conclusions given in Section 4.

## Methods

### Petri nets

A Petri net is a mathematical object based on a weighted directed bipartite graph. There are two disjoint sets of vertices called places and transitions. Places correspond to passive components of a modeled system (e.g., chemical compounds) while transitions model active components (e.g., chemical reactions or other elementary subprocesses). Places can be connected with transitions and transitions with places by arcs, which describe causal relations between elementary components of the system. The arcs are labeled by weights being positive integer numbers. Places can hold objects called tokens, which represent discrete quantities of the passive components. In a graphical representation of Petri nets places are denoted as circles, transitions as rectangles or bars, arcs as arrows and tokens as dots or positive integer numbers within places. If a weight of an arc is equal to one, usually it is not shown in the graphical representation; otherwise it is represented as a number labeling the arc.

The bipartite graph underlying a Petri net determines its structure (which should correspond to a structure of the modeled system), but most of the fundamental properties of nets of this type follow from their dynamics which is related to tokens. The flow of tokens through the net from one place to another *via* transitions corresponds to the flow of information, substances etc. through the modeled system. The distribution of tokens over all places, called marking, represents a state of the system.

The flow of tokens is governed by a transition firing rule. According to this rule, a transition is enabled if the number of tokens in each of its pre-places, i.e., the ones which directly precede this transition, is equal to at least the weight of the arc connecting such a place with the transition. An enabled transition may fire, which means that tokens flow from its pre-places to its post-places, i.e., the ones which are its immediate successors. The number of flowing tokens is equal to the weight of the arc. There are two exceptions to this rule, i.e., transitions without pre-places are continuously enabled while transitions without post-places do not produce tokens. These types of transitions can be used to model interactions of the system with its environment. A read arc is a special kind of bidirectional arc, corresponding to a pairs of arcs. Tokens in a place connected by a read arc to a transition are not consumed when firing this transition, but they are required to enable it.

Part of the results in the following paper comes from the analysis of the net simulation. In such a simulation one must set specific rules governing the firing of the transitions. Our model is based on a classical Petri net, where in general only three scenarios for transition firing are available: in a single step of the simulation only one of the enabled transitions can fire, all enabled transitions can fire simultaneously or, in a third case, only some, randomly chosen enabled transitions will fire. The latter scenario has been used in our simulations. Specifically, each enabled transition has 50% chance of firing and the sequence of enabled transitions selected for firing in a single simulation step is (each time) chosen randomly.

More complex firing scenarios could be available if, e.g., stochastic Petri net has been used. It would however require assigning a so called *firing rates* to each transition, what in general would be a difficult task requiring knowledge about more or less specific probability of each reaction involved in the modeled system. The assumption of 50% firing chance is therefore a trade-off, allowing simple yet still valuable stochastic simulations of the model.

As a summary one can say that in a single step some random enabled transitions (with 50% chance for firing) are randomly assigned to the firing list. One must note that they will not necessarily all fire, because competing transitions on such a list can share the same pre-places and consume “activation” tokens from each other depending on the firing sequence (i.e., when a transition is on that list, but because of firing of the other ones such a transition loses the necessary minimum of tokens in its pre-places, it stops being active and cannot fire). However, such a firing sequence is random in each simulation step, so a sufficient stochastic scenario is provided to study the dynamic behavior of the model.

More advanced analysis methods for simulation of a net would be available if a stochastic Petri net (SPN) or continuous Petri net (CPN) have been used to model the DNA repair process. The latter would require the formulation of ODEs for the reaction flows. For the SPN model, the so-called *firing rates* need to be assigned for each transition—in the stochastic Petri net they influence the transition chances of firing in the simulation. Both approaches have advantages (e.g., a more complex and accurate simulation) and disadvantages. As for the latter, it is still difficult to find specific and accurate values which could describe the ODE in the CPN model or firing rates for all transitions for the SPN model. Further, in a continuous Petri net an invariant, MCT and cluster-based analysis explained in the next section is not available, while in this work such an analysis allowed us to divide the Petri net model into some functional subnets, characterized by the common functionality.

### t-invariants analysis

Besides the graphical representation of Petri nets also a more formal one, called incidence matrix, is used. Entries of such matrix *A* = [*a*_*ij*_]_*n* × *m*_ are integer numbers, and entry *a*_*ij*_ is equal to a difference between numbers of tokens in places *p*_*i*_ before and after firing transition *t*_*j*_.

Among many properties of Petri nets, the ones related to t-invariants are especially important in the context of an analysis of biological systems models. A t-invariant is a vector *x* of integer numbers being a solution to the equation

A⋅x=0

For every t-invariant *x* there is a set of transitions supp(x) = {t_j_:x_j_>0,j = 1,2,…,m} called its support.

t-invariants correspond to some subprocesses of the modeled biological system which do not change its state. Hence, analyzing relations among them may lead to discoveries of properties of the system [17]. The net is covered by t-invariants if every transition belongs to a support of at least one of them. In such a situation every transition contributes to at least one process of the modeled biological system [18–20]. Therefore, when an analysis is based on t-invariants the net should be covered by them. In order to find relations between t-invariants they are grouped into sets called t-clusters, using standard clustering algorithms. Also transitions can be grouped into sets called maximal common transition sets (MCT sets) what helps in the analysis of t-invariants [17, 21]. Both t-clusters and MCT sets correspond to some functional modules of the biological system. A more precise description of a Petri net elements, invariants and MCT sets is available as Appendix in S1 Appendix.

## Results

### The model of the human base excision repair pathway

The Petri net presented in this work is a model of the human base excision repair pathway. The model was built based on data from the publicly available databases: REPAIRtoire—a database of DNA repair pathways [9], DNAtraffic [10] and Reactome [22, 23]. Subsequently, the computational model was supplemented with several new aspects of the apurinic/apyrimidinic (AP) site processing which are not included in the above-mentioned databases but were reported in the literature:

- displacement of OGG1 glycosylase at the AP site by AP endonuclease [24–26] or NEIL1 glycosylase [27, 28],
- displacement of NTH1 glycosylase by AP endonuclease [29],
- Polβ-mediated LP-BER [30, 31],
- mechanism of the repair initiated by NEIL2 and NEIL3 [32, 33].

The model has been built and analyzed using Holmes software [34] and is available in SPPED (Snoopy), XML (SMBL), .project (Holmes) and PDF formats as S1, S2, S3 and S4 Files respectively.

The model can be subdivided into 4 subnets, described below:

#### Subnet 1: Introduction of DNA damage

This part of the model represents the creation of different types of DNA damage from undamaged DNA.

#### Subnet 2: AP site formation

The biggest part of the model represents the recognition of DNA damage by DNA glycosylases, followed by the removal of damaged bases by cleavage. This part of the model can be further subdivided into 11 modules, each of which represents the activity of one DNA glycosylase (Table 1).

**Table 1:** Human DNA glycosylases studied in the Petri net model.

	Glycosylase name in the model	Biological meaning and physiological DNA substrates	Type of action: Monofunctionalor Bifunctional(β-elimination)(β,δ-elimination)	Protein family	References
1	**NTH1** (NHTL1)	**Human homolog of Endonuclease III** Tg/dsDNA; 5-OHC/dsDNA; 5-OHU/dsDNA; 5,6-diHT; FaPyG; Cg; 5,6-diOHU	Bifunctional (δ-elimination)	HhH	[10, 35–38]
2	**SMUG** (SMUG1)	**Human single-strand Selective Monofunctional Uracil-DNA Glycosylase 1** dU; 5-OHU; 5-hmU; 5-fU	Monofunctional	UDG	[10, 36, 39–41]
3	**MBD4**	**Methyl-CpG Binding Domain Endonuclease 4** 5-fU; U and T from CpG islands; 5-hmU/ssDNA, Tg opposite G, 5-hmU from U:G	Monofunctional	HhH	[10, 42, 43]
4	**MPG** (ANPG, AAG)	***N*-Methylpurine-DNA Glycosylase** 3-mA; 7-mA; *N*7-mG; *N*3-mG; 1,*N*6εA; 1,*N*(2)εG; 1-mA; 1-mG; 3-meC; Hx	Monofunctional	MPG	[10, 44–48]
5	**MYH** (MUTYH)	**MutH *E*. *coli* Homolog** A opposite 8-oxoG, C or G/ds DNA; 2-oxoA; 2-hA	Monofunctional	HhH	[10, 36, 41]
6	**NEIL1**	**Endonuclease VIII-Like 1** 5-OHC; 5-OHU/ssDNA and dsDNA; 8-oxoG opposite C, G, T; 5,6-diHT, 5,6-diOHU; FaPyA; FaPyG; FaPy7mG; Tg; DNA-psoralen, 8-oxoA opposite C	Bifunctional (β,δ-elimination)	Endonuclease VIII-like	[10, 36, 39, 49, 50]
7	**NEIL2**	**Endonuclease VIII-Like 2** 5-OHC; 5-OHU; 5,6-diHU; 5,6-diHT; 5,6-di-OHU; 8-oxoG present inside a bubble (ssDNA); FaPyA	Bifunctional (β,δ-elimination)	Endonuclease VIII-like	[10, 36, 49, 51]
8	**NEIL3**	**Endonuclease VIII-Like 3** FaPyA ssDNA; FaPyG ssDNA; 5-OHU ssDNA; 5-OHC ssDNA; Tg; 8-oxoA, spiroiminodihydantoin, guanidinohydantoin	Bifunctional (β-elimination)	Endonuclease VIII-like	[33, 36, 52]
9	**OGG1**	**8-Oxoguanine DNA Glycosylase** 8-oxoG opposite C, G, T; 8-oxoA from A:C; FaPyG; FaPy7-mG	Bifunctional (β-elimination)	HhH	[10, 36, 39, 53–55]
10	**TDG**	**T/G Mismatch-Specific Thymine DNA Glycosylase** U and T from CpG islands; 5-hmU from U:G; 5-OHC; 5-fC; 5-caC; 5-FC; 3,N(4)εC; Tg opposite G, 8-oxoA opposite C, G and T	Monofunctional	UDG	[41, 43, 56–63]
11	**UNG2** (UNG)	**Uracil-DNA Glycosylase 2 (nuclear)** UNG1 (mitochondrial) U in ssDNA and dsDNA	Monofunctional	UDG	[10, 36, 64, 65]

A–adenine; C–cytosine; G–guanine; T–thymine; U–uracil; Br–bromo; ca–carboxyl; diH–dihydro; diOH–dihydroxy; ε –etheno; f–formyl; F–fluoro; FaPy– 2,6-diamino-4-hydroxy-5-*N*-methylformamidopyrimidyne; g–glycol; Hx–hypoxanthine, m–methyl; OH–hydroxyl (h); oxo–dihydro; ss–single-stranded; ds–double-stranded.

DNA glycosylases and their substrates are represented by places. The substrates were divided into groups, based on a subset of DNA glycosylases that recognize them (Table 2).

**Table 2:** Damages to the DNA taken into account in the model.

	Group name	Damage detected	Glycosylases
1	U:G and U	U from dsDNA, U from U:G	UNG2, SMUG, TDG
2	U:A and U ssDNA	U from ssDNA, U from U:A	UNG2, SMUG
3	5-hmU:G	5-hmU from U:G	SMUG, MBD4, TDG
4	5-hmU ssDNA and 5-fU	5-hmU from ssDNA and 5-fU	SMUG, MBD4
5	5-OHU ssDNA	5-OHU ssDNA	SMUG, NEIL1, NEIL2, NEIL3
6	5-OHU paired with A/G and 5-hmU paired with A	5-OHU from U:G and U:A, 5-hmU from U:A	SMUG
7	T/U:CpG	U from U:G in CpG sites and T from T:G in CpG sites	MBD4
8	8-oxoA:C	8-oxoA from A:C	TDG, OGG1, NEIL1, NEIL2
9	5-OHC in dsDNA	5-OHC dsDNA	TDG, NTH1, NEIL1, NEIL2
10	8-oxoA paired with T/G	8-oxoA from A:T and A:G	TDG, NEIL2
11	T paired with T/C/G/O6-mG, 5-fC, 5-caC; 3,N(4)εC	T paired with T/C/G/O6-mG, 5-fC, 5-caC and 3,N(4)εC	TDG
12	FaPyG:C	FaPyG from FaPyG:C	OGG1, NTH1, NEIL1
13	A paired with G/8-oxoG/C and 2-OH/oxoA paired with T or G	A from A:G, A:8-oxoG and A:C, 2-OHA/2oxo-A from A:G	MYH
14	1/3/7-mA, 1/3/7-mG, 3-mC, 1,*N*6εA, hypoxanthine, 1,*N*(2)εG	1-mA, 3-mA, 7-mA, 1m-G, 3-mG, 7-mG, 3-mC, 1,N6εA, 1,*N*(2)εG, hypoxanthine	MPG
15	5-OHU in dsDNA; 5,6-diHT and 5,6-diOHU	5-OHU dsDNA; 5,6-diHT and 5,6-diOHU	NTH1, NEIL1, NEIL2
16	Tg paired with G	Tg from Tg:G	MBD4, TDG, NTH1, NEIL1
17	FaPyG paired with A/G/T	FaPyG from FaPyG:A, FaPyG:G and FaPyG:T	OGG1, NTH1
18	8-oxoG paired with C/G/T; FaPy-7mG	8-oxoG from G:C, G:G and G:T; FaPy-7mG	OGG1, NEIL1
19	5-OHC ssDNA	5-OHC ssDNA	NEIL1, NEIL2, NEIL3
20	Tg in ssDNA	Tg ssDNA	NEIL1, NEIL3
21	Tg paired with A, Tg in dsDNA	Tg from Tg:A, Tg dsDNA	NTH1, NEIL1, NEIL3
22	FaPyA, FaPyG, spiroiminodihydantoin, guanidinohydantoin from ssDNA	FaPyA, FaPyG, spiroiminodihydantoin and guanidinohydantoin from ssDNA	NEIL3
23	Cg from dsDNA	Cg dsDNA	NTH1
24	FaPyA	FaPyA	NEIL1, NEIL2
25	DNA-psoralen	DNA-psoralen	NEIL1
26	5,6-diHU; 8-oxoG in ssDNA	5,6-diHU, 8-oxoG ssDNA	NEIL2

**Fig 1:**
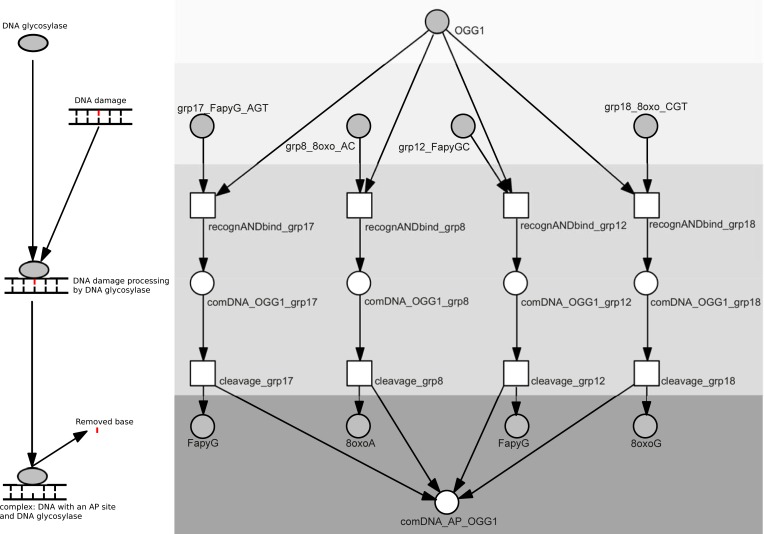
The part of the BER Petri net model presenting AP site formation by the OGG1 glycosylase. Recognition of a specific error group, the cleavage and formation of the DNA repair complex is shown for different error molecules. A general, schematic diagram of the modeled process is shown on the left. For more detailed description of places and transitions see Table 3. Shades of gray were used to mark steps of the process.

Each group of DNA damage is represented by a separate place. For each glycosylase-substrate pair this part of the model contains a transition representing the damage recognition process, which leads to the glycosylase-damaged DNA complex formation and cleavage of the damaged base, which in turn results in the AP site formation. Fig 1 presents the submodule for the OGG1 DNA glycosylase (full names of places and transitions of this submodule are given in Table 3).

**Table 3:** The biological meaning of places and transitions for Fig 1, presenting AP site formation by the OGG1 glycosylase (subnet 2).

Place / Transition Tag	Molecules / reaction represented by places and transitions
**Glycosylases**	
OGG1	OGG1
**DNA damage**	
grp8_8oxo_AC	group 8: 8-oxoA paired with C
grp12_FapyGC	group 12: FapyG paired with C
grp18_8oxoG_CGT	group 18: 8oxoG paired with C/G/T; FaPy-7mG
grp17_FapyG_AGT	group 17: FapyG paired with A/G/T
**Intermediate complexes and repair steps**	
recognANDbind_grp17	transition: OGG1 mediated recognition and binding of grp17 damage
recognANDbind_grp8	transition: OGG1 mediated recognition and binding of grp8 damage
recognANDbind_grp12	transition: OGG1 mediated recognition and binding of grp12 damage
recognANDbind_grp18	transition: OGG1 mediated recognition and binding of grp18 damage
comDNA_OGG1_grp18	place: complex: DNA with grp18 damage + OGG1
comDNA_OGG1_grp8	place: complex: DNA with grp8 damage + OGG1
comDNA_OGG1_grp12	place: complex: DNA with grp12 damage + OGG1
comDNA_OGG1_grp17	place: complex: DNA with grp17 damage + OGG1
cleavage_grp17	transition: grp17 damage cleavage by OGG1
cleavage_grp8	transition: grp8 damage cleavage by OGG1
cleavage_grp12	transition: grp12 damage cleavage by OGG1
cleavage_grp18	transition: grp18 damage cleavage by OGG1
**Products**	
comDNA_AP_OGG1	place: complex: DNA with AP site + OGG1
8oxoA	place: 8oxoA
FapyG	place: Fapy G
8oxoG	place: 8oxoG
FapyG	place: FapyG

#### Subnet 3: AP site processing

**Fig 2:**
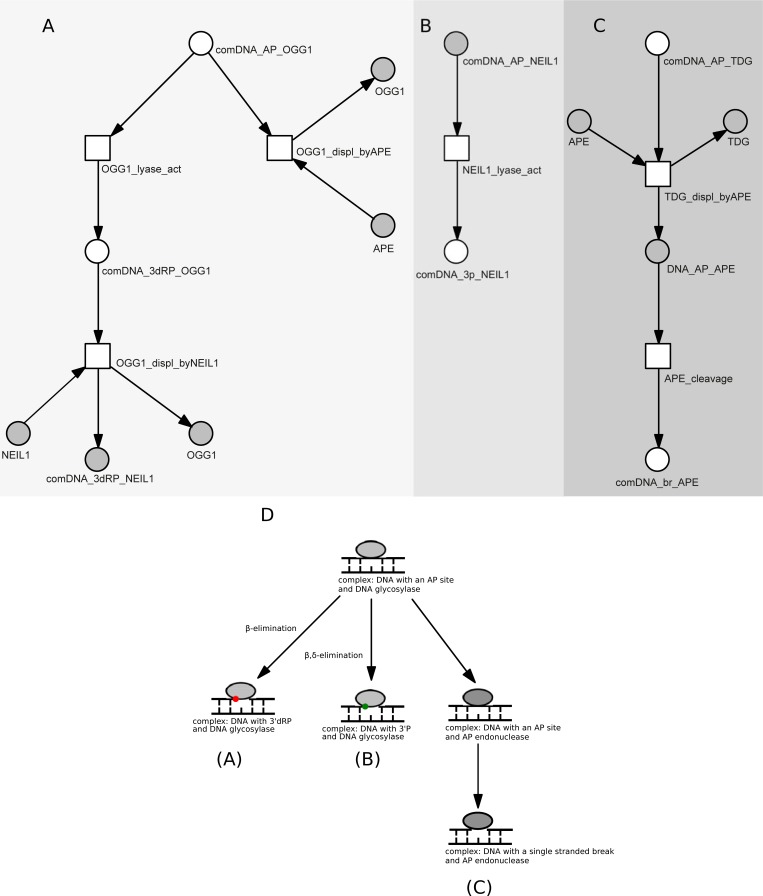
**Three parts (A-C) of the BER Petri net model presenting AP site processing by different glycosylases.** From left to right: part A—an example of the lyase activity generating 3ʹdRP end, part B—an example of the β,δ-elimination reaction, part C—an example of the DNA incision after monofunctional glycosylase activity, D—A general, schematic diagram of the modeled processes. For a more detailed description of places and transitions see Table 4. Shades of gray were used to mark different AP site processing paths.

AP site (generated by damaged base removal) is incised either by AP-lyase activity of a bifunctional DNA glycosylase or by AP endonuclease (APE). AP-lyase activities of OGG1, NTH1, NEIL1, NEIL2 and NEIL3 are represented in the model by appropriate lyase activity transitions. In the case of OGG1, NTH1 and NEIL3, which perform β-elimination, the lyase activity results in the formation of DNA incision with 3ʹdRP (deoxyribose phosphate) end (Fig 2A). Full names of places and transitions for all three Fig 2 subnets are given in Table 4.

**Table 4:** The biological meaning of places and transitions for Fig 2, presenting AP site processing by different glycosylaes (subnet 3).

Place / Transition Tag	Molecules / reaction represented by places and transitions
**Lyase activity generating 3ʹdRP end**	
comDNA_AP_OGG1	place: complex: DNA with AP site + OGG1
OGG1_lyase_act	transition: OGG1 performs β-elimination producing 3'dRP end
comDNA_3dRP_OGG1	place: complex: DNA with 3'dRP end + OGG1
OGG1_displ_byNEIL1	transition: NEIL1 stimulated displacement of OGG1
NEIL1	place: NEIL1
comDNA_3dRP_NEIL1	place: complex: DNA with 3'dRP end + NEIL1
OGG1	place: OGG1
OGG1_displ_byAPE	transition: OGG1 displacement by APE
APE	place: APE
**β,δ-elimination reaction**	
comDNA_AP_NEIL1	place: complex: DNA with AP site + NEIL1
NEIL1_lyase_act	transition: NEIL1 performs β,δ-elimination producing 3ʹP end
comDNA_3p_NEIL1	place: complex: DNA with 3ʹP end + NEIL1
**DNA incision after monofunctional glycosylase activity**	
comDNA_AP_TDG	place: complex: DNA with AP site + TDG
TDG_displ_byAPE	transition: TDG displacement by APE
TDG	place: TDG
comDNA_AP_APE	place: complex: DNA with AP site + APE
APE_cleavage	transition: cleavage of AP site by APE
comDNA_br_APE	place: complex: DNA with single strand break + APE

Only NEIL1 and NEIL2 generate DNA incision with 3ʹP end through β,δ-elimination (Fig 2B). In contrast, AP sites generated by monofunctional glycosylases (SMUG, MBD4, MPG, MYH, TDG, UDG) need additional processing and involvement of APE. This reaction results in DNA incision with 5ʹdRP end (Fig 2C).

The AP-lyase activity of OGG1 is weak ‒ after base cleavage, OGG1 can be substituted by APE, which catalyzes strand incision leading to 5ʹdRP end (Fig 2A). Similarly, APE can process AP sites generated by NTH1, and NEIL1 can catalyze β,δ-elimination at the AP sites generated by OGG1 (Fig 2A and 2C).

The 3ʹdRP and 3ʹP DNA termini (dirty ends) are refractory to DNA polymerase repair synthesis and need to be “cleaned” to 3ʹOH for BER to proceed. In human cells, APE and PNKP are responsible for the removal of 3ʹdRP and 3ʹP, respectively. 5ʹdRP end prevents the ligation process and its removal is primarily carried by DNA Polβ or FEN1.

#### Subnet 4: DNA repair synthesis and ligation

**Fig 3:**
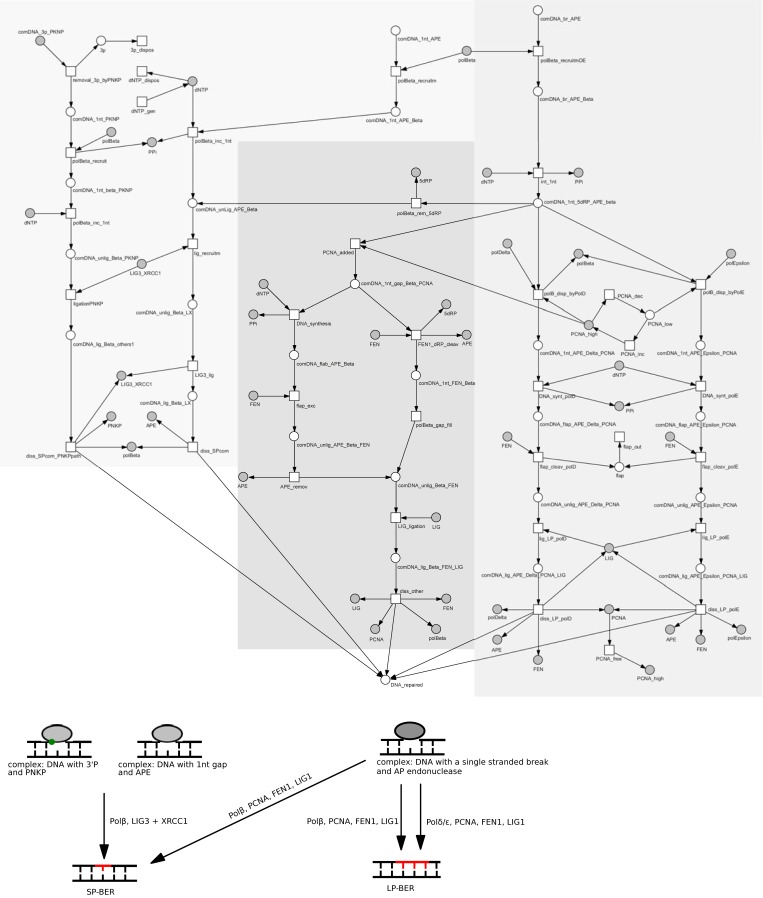
The part of the BER Petri net model presenting different repair synthesis pathways. A general, schematic diagram of the modeled process is shown below the Petri net. For more detailed description of places and transitions see Table 5. Shades of gray were used to mark different paths.

The last step of BER is the replacement of the excised nucleoside by repair synthesis catalyzed by DNA polymerases (presented in Fig 3, full names of places and transitions are given in Table 5) and nick sealing by a DNA ligase. It can proceed either as SP- or LP-BER. Typically, SP-BER is performed by Polβ and LIG3-XRCC1 complex on the products of bifunctional glycosylases activity. Repair initiated by monofunctional glycosylases proceeds *via* LP-BER which requires activity and presence of Polβ, Polδ/Polε, PCNA, FEN1 and LIG1. However, the monofunctional glycosylase pathway can also proceed *via* SP-BER, after removal of 5ʹdRP by Polβ or FEN1. If Polβ removes 5ʹdRP than the final ligation step is performed by LIG3-XRCC1 complex, otherwise it is done by LIG1. DNA synthesis in LP-BER is usually performed either by Polδ or Polε, with Polδ being preferred in high concentrations of PCNA [66]. However, it was also shown that Polβ can conduct strand synthesis in LP-BER.

**Table 5:** The biological meaning of places and transitions for Fig 3 presenting different repair synthesis pathways (subnet 4).

Place / transition Tag	Molecules / reaction represented by places and transitions
**SP-BER after bifunctional glycosylase activity**	
comDNA_1nt_APE	place: complex: DNA with 1nt gap + APE
polBeta	place: Polβ
polBeta_recruitm	transition: Polβ recruitment
comDNA_1nt_APE_Beta	place: complex: DNA with 1nt gap + APE + Polβ
polBeta_inc_1nt	transition: Polβ incorporates 1nt
dNTP	place: dNTP
dNTP_gen	transition: generate dNTP
dNTP_dispos	transition: disposal of dNTP
PPi	place: PPi
comDNA_unLig_APE_Beta	place: complex: DNA not ligated + APE + Polβ
lig_recruitm	transition: ligase recruitment
LIG3_XRCC1	place: LIG3_XRCC1
comDNA_unlig_Beta_LX	place: complex: unligated DNA + Polβ + LIG3 + XRCC1
LIG3_lig	transition: ligation byLIG3
comDNA_lig_Beta_LX	place: complex: ligated DNA + Polβ + LIG3 + XRCC1
diss_SPcom	transition: dissociation of SP-BER complex
APE	place: APE
comDNA_3p_PKNP	place: complex: DNA with3’P and + PKNP
removal_3p_byPKNP	transition: removal of3’P by PNKP
3p	place:; 3’P ending
3p_dispos	transition: disposal of 3’P
comDNA_1nt_PKNP	place: complex: DNA with 1nt gap + PNKP
polBeta_recruit	transition: Polβis recruited to the repair complex which contains 1nt gap
comDNA_1nt_Beta_PKNP	place: complex: DNA with 1nt gap + Polβ + PKNP
polBeta_inc_1nt	transition: Polβincorporates 1nt
comDNA_unlig_Beta_PKNP	place: complex: unligated DNA + Polβ + PNKP
ligationPNKP	transition: ligation by LIG3 in pathway with PNKP
comDNA_lig_Beta_others1	place: complex: ligated DNA + Polβ + LIG3_XRCC1 + PNKP
diss_SPcom_PNKPpath	transition: dissociation of SP-BER complex in pathway with PNKP
PNKP	place: PNKP
**LP-BER**	
comDNA_br_APE	place: complex: DNA with a break + APE
polBeta_recruitmDE	transition: Polβrecruitment in LP-BER
comDNA_br_APE_Beta	place: complex: DNA with a break + APE + Polβ
inc_1nt	transition: incorporation 1nt
comDNA_1nt_5dRP_APE_Beta	place: complex: DNA 1nt 5dRP + APE + Polβ
polDelta	place: Polδ
polEpsilon	place: Polε
polB_disp_byPolD	transition: Polβdisplacement by Polδ
polB_disp_byPolE	transition: Polβdisplacement by Polε
PCNA_high	place: PCNA at high levels
PCNA_low	place: PCNA at low levels
PCNA_dec	transition: PCNA decreasing
PCNA_inc	transition: PCNA increasing
comDNA_1nt_APE_Delta_PCNA	place: complex: DNA with 1nt gap + APE + Polδ + PCNA
comDNA_1nt_APE_Epsilon_PCNA	place: complex: DNA with 1nt gap + APE + Polε + PCNA
DNA_synt_polD	transition: DNA synthesis (LP) by Polδ
DNA_synt_polE	transition: DNA synthesis (LP) by Polε
comDNA_flap_APE_Delta_PCNA	place: complex: DNA with a flap + APE + Polδ + PCNA
comDNA_flap_APE_Epsilon_PCNA	place: complex: DNA with a flap + APE + Polε + PCNA
FEN	place: FEN1
flap_cleav_polD	transition: flap cleavage (LP) by Polδ
flap_cleav_polE	transition: flap cleavage (LP) by Polε
flap	place: flap
flap_out	transition: flap out
comDNA_unlig_APE_Delta_PCNA	place: complex: DNA with an unligated patch + APE + Polδ + PCNA
comDNA_unlig_APE_Epsilon_PCNA	place: complex: DNA with an unligated patch + APE + Polε + PCNA
lig_LP_polD	transition: ligation by LIG1 in LP-BER with Polδ
lig_LP_polE	transition: ligation by LIG1 in LP-BER with Polε
LIG	place: LIG1
comDNA_lig_APE_Delta_PCNA_LIG	place: complex: ligated DNA + APE + Polδ + PCNA + LIG1
comDNA_lig_APE_Epsilon_PCNA_LIG	place: complex: ligated DNA + APE + Polε + PCNA + LIG1
diss_LP_polD	transition: dissociation of LP-BER complex with Polδ
diss_LP_polE	transition: dissociation of LP-BER complex with Polε
PCNA	place: PCNA
PCNA_free	transition: PCNA freed from DNA complex after repair
**LP-BER with polBeta and SP-BER with FEN**	
polBeta_rem_5dRP	transition: Polβremoves 5ʹdRP
5dRP	place: 5’dRP
PCNA_added	transition: PCNA added to the complex
comDNA_1nt_gap_Beta_PCNA	place: complex: DNA with 1nt gap and 5ʹdRP + APE + Polβ + PCNA
DNA_synthesis	transition: DNA synthesis by Polβ
comDNA_flap_APE_Beta	place: complex: DNA with a flap structure + APE + Polβ
flap_exc	transition: flap excision
comDNA_unlig_APE_Beta_FEN	place: complex: unligated DNA + APE + Polβ + FEN1
APE_remov	transition: APE removal
FEN1_dRP_cleav	transition: FEN1 5ʹdRP cleavage
comDNA_1nt_FEN_Beta	place: complex: DNA with 1nt gap + FEN1 + Polβ
polBeta_gap_fill	transition: Polβgap filling
comDNA_unlig_Beta_FEN	place: complex: unligated DNA + Polβ + FEN1
LIG_ligation	transition: ligation by LIG1
comDNA_lig_Beta_FEN_LIG	place: complex: ligated DNA + Polβ + FEN1 + LIG1
diss_other	transition: dissociation of repair complex
DNA_repaired	place: repaired DNA after damage

## Mathematical analysis of the model

The presented Petri net model consists of 259 transitions and 179 places. Full names of places and transitions are given in Tables A and B in S1 Tables, respectively.

In the first step of the analysis of the model, some of its structural properties have been identified. The net is pure and ordinary, i.e., there are no read arcs, and all the arcs have a weight equal to 1. Therefore, the net is homogenous–all outgoing arcs of a given place have the same weight. The network is connected, but not strongly connected, meaning that all its nodes have non-directed connection(s) with the others, but there are pairs of nodes without a directed path between them. The net is not conservative, i.e., many transitions consume a different number of tokens from their pre-places than they produce in the post-places. There are also static conflicts–some transitions share the same pre-places. Finally, there are input and output transitions in the model, which correspond to the connections of the analyzed system with other processes.

An important part of the analysis of the net is based on the t-invariants. There are 245 t-invariants and the net is covered by them. In the presented model of DNA BER-repair process, there are 94 non-trivial MCT sets, i.e., containing more than one transition. Many of the MCT sets are responsible for almost the same functionality, but operating on different substrates. Description of the most important MCT sets is presented in Table 6.

**Table 6:** Maximum Common Transitions sets and their biological functions.

MCT sets	Contained transitions	Biological meaning
m_1_	t_90_, t_93_, t_94_, t_95_, t_97_, t_98_	Polβ in SP-BER using PNKP.
m_2_	t_0_, t_47_, t_49_, t_58_, t_60_	Polδ in LP-BER.
m_3_	t_1_, t_48_, t_50_, t_59_, t_61_	Polε in LP-BER.
m_4_-m_7_, m_9_-m_12_, m_18_, m_21_-m_37_, m_41_-m_62_-m_94_	from 2 to 4 transitions at maximum	Detection of DNA damage by a proper glycosylase connected with the disposal of by-products. Also processes creating crucial polypeptides of the system.
m_8_	t_40_, t_169_, t_170_, t_171_	DNA incoming and leaving the repair process.
m_13_	t_32_, t_41_, t_90_	Cleavage of AP site by APE connected with Polβ recruitment to the DNA-proteins complex.
m_14_	t_42_, t_62_, t_85_	Removal of 3ʹdRP by APE, Polβ recruitment leading to SP-BER only.
m_15_	t_46_, t_56_, t_57_	XRCC1 and LIG3 ligase recruitment, SP-BER with DNA-Polβ complex followed by complex dissociation.
m_16_	t_102_, t_108_, t_109_	PCNA added to DNA-Polβ-APE complex, ligation and dissociation of the complex.
m_17_	t_103_, t_105_, t_107_	LP-BER (sub-path parallel to m_39_ MCT)
m_19_	t_6_, t_87_	NEIL2 AP-lyase activity
m_20_	t_8_, t_9_	NEIL3 AP-lyase activity
m_38_	t_52_, t_53_	NTH1 AP-lyase activity
m_39_	t_99_, t_177_	NEIL1 AP-lyase activity on 3ʹdRP
m_40_	t_100_, t_106_	FEN1 cleavage and Polβ gap filling (sub-path parallel to m_15_ MCT).
35 trivial MCT sets	single transition	Various auxiliary functions within the base excision repair DNA process not covered by MCT m_1_-m_89_.

The most important basic functions of the modeled system correspond to eight MCT sets: m_1_ (short patch repair with Polβ and PNKP), m_14_ followed by m_15_ (short patch repair for DNA-APE complex), m_16_, m_17_, m_40_ (short patch (SP) and long patch (LP) repair in which Polβ and FEN1 are involved), finally m_2_ and m_3_ responsible for long patch repair using Polδ and Polε, respectively. Two very important sets are m_13_ and m_14_. The latter is crucial for starting one of the short patch repairs with Polβ. Set m_13_ is responsible for the preparation of the DNA complex for four out of six repair paths, including three long patch repairs (with Polβ, Polδ, and Polε). Overall, the results of analysis of the MCT sets suggest that in most cases the chosen repair sub-pathway is determined by the damage type at the very beginning of the process. However, in some cases alternative pathways are available. For example repair processes in which generation of 5′dRP by APE activity takes place, the repair can either proceed via SP-BER (m15) or via LP-BER, if PCNA level is high (m17, m40).

The next step of the model analysis concerns t-clusters computation. Such successfully obtained clusters can divide the Petri net into different subnets responsible for the similar or common functions in the whole DNA repair process. Such a division is based on the t-invariants set. The description of such subnets/clusters can use the previously computed MCT sets for simplification (by replacing some groups of transitions by such sets), yet one should have in mind that such a t-cluster analysis is based solely on the computed t-invariants sets.

In order to find the optimal number of clusters, many similarity measures and grouping algorithms had to be used and their results compared. The theory is that for each cluster (group) its t-invariants must be very similar to each other according to the chosen similarity measure, and as much as possible dissimilar to t-invariants belonging to different groups [67]. Three important choices needed to be made: choosing the proper similarity measure, the clustering algorithms and the number of clusters. Thorough comparative tests of different choices in these three variables have been performed. In the tests eight similarity measures have been used: Binary, Canberra, Euclidean, Manhattan, Maximum, Minkowski, uncentered and centered Person (the latter often called Correlated Pearson metric). Further, seven clustering algorithms have been tested: Centroid, Complete, McQuitty, Median, Single, UPGMA, and Ward. The evaluation concerned the number of clusters ranging from 2 to 30. In order to evaluate the results two indices have been used, i.e., Mean Split Silhouette (MSS) [68] and Caliński-Harabasz index [69]. For the presented Petri net the best clustering has been obtained using the UPGMA algorithm and correlated Pearson similarity measure. The best number of clusters according to the used evaluation measures is 27. However, 20 of them are trivial single t-invariants responsible for the fluctuations of the system components, like APE, Polβ, Polδ, Polε and the glycosylases responsible for damage detection. The obtained clustering (i.e., set of clusters) is presented in Table 7.

**Table 7:** Clusters and their biological interpretations.

Cluster	Cluster size:	Biological interpretation
1–20	1 t-inv. per cluster	APE, PCNA, other polymerases and glycosylases fluctuations in the model
21	24	Short patch repair using Polβ and PNKP for OGG1, NEIL1 and NEIL2
22	16	Short patch repair using Polβ, APE, LIG3 and XRCC1 for OGG1, NEIL3, NTH1
23	41	Long patch repair using Polβ, PCNA, with flap excision using FEN1 and ligation with LIG1 for all glycosylases except NEIL1 and NEIL2
24	37	Short patch repair after 5ʹdRP removal by Polβ for all glycosylases except NEIL1, NEIL2
25	37	Long patch repair using Polβ, then Polδ for all glycosylases except NEIL1 and NEIL2
26	37	Long patch repair using Polβ, then Polε for all glycosylases except NEIL1 and NEIL2
27	33	Short patch repair using Polβ, PCNA, FEN1 cleavage, Polβ gap filling and final ligation with LIG1 for all glycosylases except NEIL1 and NEIL2

Clusters 21–27 from Table 7 are all responsible for different repair paths within the presented net. Table 8 presents a more detailed description of each path, providing information about e.g., glycosylases involved in damage detection or main polymerases involved in the repair process.

**Table 8:** Clusters and repair paths.

Cluster	Repair	Glycosylase	Comment	Polymerase	Other proteins
21	SP_(1)_ (*PNKP)*	OGG1, NEIL1, NEIL2	NEIL1 or NEIL2 displaced by PNKP, OGG1 first displaced by NEIL1 then NEIL1 by PNKP	Polβ	PNKP, LIG3, XRCC1
22	SP_(2)_ (*APE*)	OGG1, NEIL3, NTH1	glycosylase displacement after lyase activity	Polβ	APE, LIG3, XRCC1
23	LP (Polβ) (*PCNA*, *path 1*)	all except NEIL1 and NEIL2	all glycosylases displaced by APE	Polβ	APE, PCNA, LIG1, FEN1
24	SP_(3)_ (*APE*)	all except NEIL1 and NEIL2	all glycosylases displaced by APE	Polβ	APE, LIG3, XRCC1
25	LP (Polδ)	all except NEIL1 and NEIL2	all glycosylases displaced by APE	Polβ, Polδ	APE, PCNA, LIG1, FEN1
26	LP (Polε)	all except NEIL1 and NEIL2	all glycosylases displaced by APE	Polβ, Polε	APE, PCNA, LIG1, FEN1
27	SP_(4)_ (*PCNA*, *path 2*)	all except NEIL1 and NEIL2	all glycosylases displaced by APE	Polβ	APE, FEN1, LIG1

Table 9 contains the names of the most important transitions corresponding to the elementary processes within short and long patch repair pathways.

**Table 9:** Repair pathways construction.

Repair pathway	Simplified sequence of transitions (reactions) after detection and glycosylase removal
SP_(1)_ (*PNKP*, *cluster 21*)	NEIL1(2)_displacement, removal_3P_by_PNKP, disposal_of_3P, Polβ_recruitment_and_1nt_incorp_with_PNKP, Polβ_incorporates_1nt_PKNP_path, ligation_for_PNKP, dissociation_SP_complex_PKNP_path
SP_(2)_ (*APE*, *cluster 22*)	removal_3dRP_by_APE, Polβ_recruitment, Polβ_incorporates_1nt, ligase_recruitment, ligation_LIG3, dissociation_SP_complex
LP_(1)_ (*APE*, *PCNA path 1*, *cluster 23*)	cleavage_of_APs_by_APE, Polβ_recruitment, incorporation_1nt, PCNA_added_to_the_complex, DNA_synthesis, flap_excision, APE_removal, ligation_LIG1, dissociation
SP_(3)_ (*APE*, *cluster 24*)	cleavage_of_APs_by_APE, Polβ_recruitment, incorporation_1nt, Polβ_removes_5dRP, ligase_recruitment, ligation_LIG3, dissociation_SP_complex
LP_(2)_ (Polδ, *cluster 25*)	cleavage_of_APs_by_APE, Polβ_recruitment, incorporation_1nt, Polβ_displacement_by_ Polδ, DNA_synthesis_LP_Polδ, flap_cleavage_LP_Polδ, ligation_in_LP_Polδ, dissociation_LP_Polδ_complex
LP_(3)_ (Polε, *cluster 26*)	cleavage_of_APs_by_APE, Polβ_recruitment, incorporation_1nt, Polβ_displacement_Polε, DNA_synthesis_LP_Polε, flap_cleavage_LP_Polε, ligation_in_LP_Polε, dissociation_LP_ Polε_complex
SP_(4)_ (*FEN1*, *cluster 27*)	cleavage_of_APs_by_APE, Polβ_recruitment, incorporation_1nt, PCNA_added_to_the_complex, FEN1_dRP_cleavage, Polβ_gap_filling, ligation_LIG1, dissociation

### Analysis of the robustness of the BER pathway *via* systematic *in silico* knockout experiment

The impact of each modeled repair synthesis sub-paths: three short-patch (SP) ones (where DNA Polβ is always present) and three long-patch (LP) repairs (with polymerases δ, ε or β) were analyzed *via* systematic *in silico* knockout experiment. The six repair synthesis sub-paths defined for this analysis are as follows:

- Path 1: SP_(1)_, refers to t-invariants cluster 21, DNA synthesis performed by Polβ, includes PNKP,
- Path 2: SP_(2)_, refers to t-invariants cluster 22, DNA synthesis performed by Polβ,
- Path 3: LP_(1)_, refers to t-invariants cluster 23, DNA synthesis performed by Polβ,
- Path 4: SP_(4)_, refers to t-invariants cluster 27, DNA synthesis performed by Polβ, includes FEN1,
- Path 5: LP_(2)_, refers to t-invariants cluster 25, DNA synthesis performed by Polδ,
- Path 6: LP_(3)_, refers to t-invariants cluster 26, DNA synthesis performed by Polε.

Net simulations were used to perform a series of knockout experiments. Each simulation contained 20,000 steps and was repeated 20 times. In a single step, any enabled transition had a 50% chance for firing. Transition t_169_ (DNA_back_to_pool) was treated as a marker of successful DNA repair. Its chance of firing represents the chance of successful completion of the repair process. The results of the simulations for the non-disturbed model are summarized in Table 10. The chance of successful repair, represented by the firing of transition t_169_, equals on average 23.3%. The biggest proportion of successful repairs is processed *via* the SP_(2)_ pathway.

**Table 10:** Involvement of the synthesis sub-paths in the successful DNA repair in the non-disturbed model.

Repair synthesis sub-path	Transition	Chances for firing
Path 1 (SP)	t_95_: dissociation_SP_complex_PKNP_path	6.8% (0.068)
Path 2 (SP)	t_46_: dissociation_SP_complex	12.9% (0.129)
Path 3 (SP)	t_107_: APE_removal	0.3% (0.003)
Path 4 (SP)	t_106_: Polβ_gap_filling	1.2% (0.012)
Path 5 (LP)	t_47_: dissociation_LP_Polδ_complex	1.2% (0.012)
Path 6 (LP)	t_48_: dissociation_LP_ Polε_complex	1.3% (0.013)

For each repair synthesis sub-path, the chance of firing of the transition directly preceding transition t_169_ is shown. Chances of firing were averaged from 20 simulations.

The results of the knockout experiments are presented in Table 11. Transitions critical for each repair synthesis sub-paths have been identified, and their impact on the overall chance of successful repair was checked. The most prominent change in the repair success rate was observed when Path 2 was disabled. In this setup Paths 1 and 3–6 exhibit a slightly higher activity but they cannot fully recapitulate the repair. At the same time the lack of activity in Paths 3–6 did not result in an observable change in DNA repair efficiency. The high importance of Polβ in DNA repair is a well-known phenomenon—the lower repair rate is thus expected (see Discussion for more details). To the best of our knowledge, Polδ and Polε knockouts influence on BER efficiency and higher activity of other BER sub-pathways after Polβ knockout have not been reported yet.

**Table 11:** Results of the *in silico* knockout experiments.

Path disabled	Transitions disabled	Other paths frequency impact	Chance of successful repair
Path 1	t_90_: removal_3P_by_PNKP	Path 1: t_95_: disabled Path 2: t_46_: 0.130 (+0.001) Path 3: t_107_: 0.004 (+0.001) Path 4: t_106_: 0.013 (+0.001) Path 5: t_47_: 0.012 Path 6: t_46_: 0.013	0.169 (- 0.064)
Path 2 (1 of 2 starting points)	t_62_: Polβ_recruitment	Path 1: t_95_: 0.071 (+0.003) Path 2: t_46_: 0.080 (-0.049) Path 3: t_107_: 0.004 (+0.001) Path 4: t_106_: 0.013 (+0.001) Path 5: t_47_: 0.012 Path 6: t_46_: 0.013	0.190 (- 0.043)
Path 2 (completely)	t_62_: Polβ_recruitment t_96_: Polβ_removes_5dRP	Path 1: t_95_: 0.068 Path 2: t_46_: disabled Path 3: t_107_: 0.008 (+0.005) Path 4: t_106_: 0.029 (+0.017) Path 5: t_47_: 0.028 (+0.016) Path 6: t_46_: 0.014 (+0.001)	0.141 (- 0.092)
Paths 3 **and** 4	t_102_: PCNA_added_to_the_complex	Path 1: t_95_: 0.068 Path 2: t_46_: 0.134 (+0.005) Path 3: t_107_: disabled Path 4: t_106_: disabled Path 5: t_47_: 0.017 (+0.005) Path 6: t_46_: 0.015 (+0.002)	0.233 (no impact)
Path 3	t_103_: DNA_synthesis	Path 1: t_95_: 0.068 Path 2: t_46_: 0.128 (-0.001) Path 3: t_107_: disabled Path 4: t_106_: 0.012 Path 5: t_47_: 0.012 Path 6: t_46_: 0.013	0.233 (no impact)
Path 4	t_100_: FEN1_dRP_cleavage	Path 1: t_95_: 0.068 Path 2: t_46_: 0.131 (+0.002) Path 3: t_107_: 0.011 (+0.008) Path 4: t_106_: disabled Path 5: t_47_: 0.011 (-0.001) Path 6: t_46_: 0.012 (-0.001)	0.232 (no impact)
Path 5 **and** 6	t_60_: Polβ_displacement_by_Polδ t_61_: Polβ_displacement_by_Polε	Path 1: t_95_: 0.070 (+0.002) Path 2: t_46_: 0.142 (+0.013) Path 3: t_107_: 0.006 (+0.003) Path 4: t_106_: 0.023 (+0.011) Path 5: t_47_: disabled Path 6: t_46_: disabled	0.234 (no impact)
Path 5	t_60_: Polβ_displacement_by_Polδ	Path 1: t_95_: 0.068 Path 2: t_46_: 0.132 (+0.003) Path 3: t_107_: 0.005 (+0.002) Path 4: t_106_: 0.018 (+0.006) Path 5: t_47_: disabled Path 6: t_46_: 0.015 (+0.002)	0.234 (no impact)
Path 6	t_61_: Polβ_displacement_by_Polε	Path 1: t_95_: 0.068 Path 2: t_46_: 0.135 (+0.006) Path 3: t_107_: 0.004 (+0.001) Path 4: t_106_: 0.015 (+0.002) Path 5: t_47_: 0.015 (+0.003) Path 6: t_46_: disabled	0.234 (no impact)

Transitions found to be critical for each repair synthesis sub-path are listed together with their impact on other synthesis sub-paths. Chances of successful repair were averaged from 20 experiments. The change compared to non-disturbed model is also given in parenthesis (if the change has been at least 0.001 or more).

We have also investigated influence of a glycosylase knockout on the modeled behavior of BER, taking the TDG glycosylase as an example. We found that TDG knockout, simulated by inactivation of transition t_206, which introduces TDG into the system, leads to: i) higher activity of glycosylases involved in processing of the same DNA damage substrates as TDG (namely MBD4, OGG1, NEIL1, NEIL2 and SMUG), ii) slight decrease in the overall DNA repair efficiency, iii) accumulation of DNA damage from group 11, which are recognized only by TDG and iv) decreased activity of the SP-BER pathway.

## Discussion

The BER process can be described as a unique DNA repair pathway, where very precise enzymes are used to recognize and remove specific DNA damage. Thus far eleven DNA glycosylases have been found in human cells (Table 1). All DNA glycosylases are divided into four structurally different superfamilies and share a similar ability to recognize the DNA damage. However, there are also several mismatches (e.g., T-C) or DNA damage types (e.g., 3-mA, 7-mG) which are removed only by one human enzyme (Table 2) [9, 10, 36, 65, 70].

Although BER has already been a subject of several computational modeling attempts, we provide the most comprehensive model, which includes the widest range of protein factors involved in the DNA repair process and the biggest collection of DNA damage types (Table 1). The models available to date concentrate mainly on the repair initiated by the OGG1 glycosylase [12–14], while our computational model of human BER includes 68 different DNA damage types found in living cells and identified and repaired by 11 human DNA glycosylases. We have also included into our Petri net model several mechanisms that have not been considered in the available models, e.g., displacement of OGG1 at the AP site by AP endonuclease or NEIL1, displacement of NTH1 by AP endonuclease, Polβ-mediated LP-BER and the mechanism of the repair initiated by NEIL2 and NEIL3. However, we have included in our model mainly the major DNA damage types. As a consequence, some of the products of minor G base oxidative damage (e.g. 5-carboxamido-5-formamido-2-iminohydantoin, 2Ih) which can be removed from DNA by hNEIL1-3 glycosylases [71] are not present in the model. We have also omitted several bulky DNA damage products (from the minor fraction) which are used mostly for *in vitro* studies of DNA glycosylases activities.

BER can proceed *via* different sub-pathways, depending on the damage and the type of DNA glycosylase that detected the problem (Table 2). In general, the repair DNA synthesis can be performed by DNA Polβ (Polβ, used for both the so-called short-patch (SP) and long-patch (LP) base excision repairs). Two other major DNA polymerases δ and ε (Polδ and Polε) can repair DNA instead of Polβ, and they are always involved in the LP-BER pathway with PCNA involvement. Other possibilities for repairing DNA by the BER mechanism have also been included in our model. The analysis performed on the base of net t-invariants allowed to divide a structure of the net into meaningful biological units (MCT sets) and more importantly to group t-invariants which represent basic subprocesses of the model into clusters (Table 6). The clusters allow for separating different repairing processes, showing the glycosylases, polymerases and other compounds involved in the repair processes, as presented in Tables 7 and 8 and Figs 1–3.

Using *in silico* simulations, we showed that most efficient and successful DNA repair of the studied DNA damage was reached *via* SP-BER (Table 10). This result was consistent with biological data showing that the patch size in the nucleosomal BER was rather shorter (1 nt) than longer (2–12 nt) [72]. Our *in silico* knockout experiments showed that Polβ, the so-called repair polymerase (Table 11, Path 2) is necessary for BER. Lack of DNA Polβ in our model resulted in stopping the cleaning of 5’dRP moiety and gap filling (DNA synthesis) which was simultaneously manifested as a block of whole SP-BER. In living cells, the perturbation in BER can lead to genomic instability. Our results could be supported by the observation that somatic mutations in *Polβ* gene have been detected in various types of cancers (NCI Genomic Data Commons (GDC)). However, it is still not clear whether and how Pol*β* mutations and overexpression can be linked to cancer onset and its progression. Recently, it has been shown that human R152C Pol*β* mutant was impaired in BER activity and efficiency. Moreover, the mutant cells have displayed a high frequency of chromatid breakages [73]. Mice carrying a targeted disruption of the *Polβ* gene has shown growth retardation and died of a respiratory failure immediately after the birth [74]. Also, homozygous Pol*β* R137Q knock-in mice embryos were typically small in size and had a high mortality rate (21%). In this mutant the BER efficiency was impaired, which subsequently ended in double-strand breaks (DSBs) and chromosomal aberrations [75]. Recently it has been shown that a mouse model with decreased expression of *Polβ* (with Y265C mutation) developed systemic lupus erythematosus (SLE) [76].

It is not clear which of the two activities of Pol*β* is the most important in SP-BER. Both DNA synthesis and 5′-dRP-lyase activity can be replaced by other DNA polymerases or FEN1, respectively. If the 5′-dRP moiety is reduced or oxidized, the 5′-dRP-lyase of Pol*β* cannot remove the modified sugar residue, and LP-BER is initiated. Next, a flap (2–10 nucleotide long) is subsequently removed by FEN1. FEN1 mutations are very rare, suggesting that FEN1 is important for normal DNA metabolism [77, 78]. Targeted deletion of the *Fen1* gene in mice causes early embryonic lethality [79]. However, several somatic FEN1 mutations have been detected in human cancer (GDC) but the relationship between FEN1 deficiency and cancer susceptibility remains unclear. Recently, it was reported that L209P FEN1 mutation is associated with colorectal cancer. Human L209P FEN1 mutant was lacking the exo- and endonuclease activities but retaining DNA-binding affinity. This was a dominant-negative mutation and mutated protein impaired LP-BER *in vitro* and *in vivo* [80]. In contrast, our computational Petri net BER-knockout model with eliminated FEN1 activity did not show any significant change in BER efficiency (Table 11, Path 4). This inconsistency can be explained by the fact that FEN1 functions not only in LP-BER and it is mostly recognized as a central component of cellular DNA metabolism (e.g. processing of Okazaki fragment maturation intermediates, telomere maintenance and rescue of stalled replication fork) [77].

It is estimated that each day as many as 10,000 abasic sites are formed in one human cell [81]. Removing AP-sites from DNA is a daily task for the DNA repair/tolerance system. Apurinic/apyrimidinic endonuclease 1 is responsible for the AP sites processing which is necessary for further steps of DNA repair pathways [82, 83]. In our computational BER model, the elimination of AP-sites endonuclease 1 (APE1) resulted in downstream blocking of DNA repair, both, SP-BER and LP-BER. Lack of AP-sites repair can be an explanation of the embryonic lethality of Ape1 null mice [84, 85] and increasing tumor susceptibility in heterozygous mice [86]. APE1 mutations in humans (APE1 variants: L104R, E126D, and R237A, exhibiting approximately 40–60% reduction in specific incision activity) have been associated with amyotrophic lateral sclerosis (ALS) [87, 88]. Also, somatic APE1 mutations (APEX P112L, W188X, and R237C) were found in endometrial cancers [89].

Preparation of the 3′OH ends is a key step for repair DNA synthesis. In our model, we observed a reduction of a chance for successful repair when polynucleotide kinase/phosphatase (PNKP) was removed from the pathway (Table 11, Path 1). PNKP involvement in BER is mostly due to APE-independent base excision repair pathway in human cells after NEIL1 and NEIL2 action on oxidized base lesions [27, 28]. However, PNKP (two activities: DNA 5'-kinase and DNA 3′-phosphatase) is mostly recognized as an enzyme which generates 5′-phosphate/3′-hydroxyl DNA termini that are critical for ligation by the non-homologous end joining (NHEJ) DNA ligase LigIV during double-strand break repair (DSBR). Microcephaly with early-onset, intractable seizures and developmental delay (MCSZ) is a hereditary disease caused by mutations in PNKP [90, 91].

Eleven human DNA glycosylases were *in silico* deleted one-by-one in our computational BER model. None of them was critical for BER function because almost each DNA damage can be removed by at least two distinct glycosylases (Table 2). Apart from DNA damage removal, various BER glycosylases (SMUG, MBD4, TDG, UNG2, NEIL1-3) are involved in nucleotide replacement during the active DNA demethylation process [57]. However, unlike other DNA glycosylases, TDG is essential for embryonic development; mice die at day 11.5 [92]. The lethal phenotype is associated with epigenetic aberrations affecting the expression of developmental genes. Mouse embryonic fibroblasts, (MEFs) derived from Tdg null embryos showed impaired gene regulation due to imbalance histone modification and CpG methylation [93]. Other DNA glycosylases are not essential, mouse knockouts are viable, however, showing increase in mutation frequency or some immune dysfunction [36]. Human mutant DNA glycosylases have also been widely described and correlated with a predisposition to various diseases [70]. Also, an interplay between DNA repair of the oxidatively damaged base, 8-oxoG (8-oxo-7,8-dihydroguanine) and transcriptional activation has been documented for mammalian genes: removal of 8-oxoG from the coding strand by OGG1-mediated BER resulted in upregulated transcription [94]. Since oxidation is ongoing and transition of C to U occurs spontaneously or at specific times during differentiation and development, there is a strong suggestion that BER substrates might be epigenetic and modulate transcription factor binding [71, 95].

We believe that our model can be a valuable tool that can help to predict the influence of changes in protein activities or levels on the repair process as well as some regulatory functions. It can be used to predict the sensitivity of the cell with inactivated repair proteins to different types of DNA damage and it can help to identify the by-passing pathways that may lead to lack of pronounced phenotypes associated with mutations in some of the proteins.

## Supporting information

S1 Tables Two supplementary Tables A–B listing all net places and transitions.
(PDF)

S1 Appendix Appendix with more detailed description of Petri net elements and theory. (PDF)

S1 File The model in SPPED format. (SPPED)

S2 File The model in SMBL format. (XML)

S3 File The model as Holmes project. (PROJECT)

S4 File The model as a figure in PDF format. (EPS)
