# Supplementary material for: Petri net–based model of the human DNA base excision repair pathway
Source: PLoS One. 2019 Sep 13;14(9):e0217913. doi: 10.1371/journal.pone.0217913 (PMC6743755; doi:10.1371/journal.pone.0217913)
Supplement: S1 Tables — Two supplementary Tables A–B listing all net places and transitions. (PDF) [file pone.0217913.s001.pdf]

Table A. Biological meaning of all Petri net places.

Place	Biological meaning
p_0	APE
p_1	DNA_1nt_APE_polDELTA_PCNA
p_2	DNA_1nt_APE_polEPSILON_PCNA
p_3	DNA_APsite_with_APE
p_4	complex_DNA_break_APE_BETA
p_5	DNA_flap_APE_poEPSILON_PCNA
p_6	DNA_flap_APE_polDELTA_PCNA
p_7	complex_DNA_3dRP_NEIL3
p_8	complex_DNA_3dRP_hNTH1
p_9	DNA_ligated_APE_polDELTA_PCNA_LIG1
p_10	DNA_ligated_APE_polEPSILON_PCNA_LIG1
p_11	DNA_unligpatch_APE_pEPSILON_PCNA
p_12	DNA_unligpatch_APE_polDELTA_PCNA
p_13	complex_DNA_3dRP_OGG1
p_14	FEN1
p_15	LIG1
p_16	LIG3_XRCC1
p_17	MBD4
p_18	MPG
p_19	NEIL1
p_20	NEIL3
p_21	OGG1
p_22	PCNA
p_23	PCNA_high_level
p_24	PCNA_low_level
p_25	PNKP
p_26	PPi
p_27	TDG
p_28	UNG2
p_29	group14_waste
p_30	_8oxoAC
p_31	compleDNA_UNG2_5hydroUra
p_32	complexDNA_APs_MBD4
p_33	complexDNA_APs_UNG2
p_34	complexDNA_APs_hNTH1
p_35	complexDNA_APs_hSMUG1
p_36	complexDNA_MBD4_5hmU_ssDNA
p_37	complexDNA_MYH_gr13
p_38	complexDNA_TDG_5OHC_dsDNA
p_39	complexDNA_TDG_8oxoAC
p_40	complexDNA_TDG_uracil
p_41	complexDNA_UNG2_uracil
p_42	complexDNA_hNTH1_FapyG

p_43	complexDNA_hNTH1_Cg_dsDNA
p_44	complexDNA_hNTH1_5OHC_dsDNA
p_45	complexDNA_hNTH1_thymGlycol
p_46	complexDNA_hSMUG1_uracil
p_47	complex_DNA_APs_MYH
p_48	complex_DNA_1nt_5dRP_APE_polBETA
p_49	complex_DNA_APs_NEIL2
p_50	complex_DNA_APs_NEIL1
p_51	complex_DNA_APs_NEIL3
p_52	complex_DNA_MPG_gr14
p_53	complex_DNA_APs_TDG
p_54	complex_DNA_APs_OGG1
p_55	complex_DNA_MPGbound_AP_site
p_56	complex_DNA_NEIL1_5hydroxyU
p_57	complex_DNA_NEIL1_8oxyG
p_58	complex_DNA_NEIL1_FaPyGC
p_59	complex_DNA_NEIL1_thymineGlicol
p_60	complex_DNA_NEIL2_5hydroxyU
p_61	complex_DNA_NEIL3_5hydroxyU
p_62	complex_DNA_NEIL3_FaPyA_FaPyG_other
p_63	complex_DNA_NEIL3_Tg_in_dsDNA
p_64	complex_DNA_OGG1_8oxoG
p_65	complex_DNA_OGG1_FaPyGC
p_66	complex_DNA_with_1nt_gap_APE
p_67	complex_DNA_with_1nt_gap_APE_polBETA
p_68	complex_DNA_not_ligated_APE_polBeta
p_69	complex_DNA_ligated_polBETA_LIG3_XRCC1
p_70	complex_DNA_unligatedres_polBETA_LIG3_XRCC1
p_71	dNTP
p_72	_5dRP
p_73	MYH
p_74	FaPyGC
p_75	NTH1
p_76	SMUG
p_77	repaired_DNA
p_78	polDELTA
p_79	polEPSILON
p_80	polBETA
p_81	_5OHC_ssDNA
p_82	uracil
p_83	complex_DNA_3dRP_APE
p_84	complex_DNA_3P_NEIL2
p_85	complex_DNA_3P_NEIL1
p_86	APE_high_level
p_87	complex_DNA_3P_PKNP
p_88	flap

p_89	complex_DNA_break_APE
p_90	complex_DNA_1nt_gap_PNKP
p_91	complex_DNA_unligated_polBETA_PNKP
p_92	complex_DNA_ligated_polBETA_LIG3_XRCC1_PNKP
p_93	_3P
p_94	complex_DNA_with_1nt_gap_polBETA_PKNP
p_95	APE_low_level
p_96	g1_U_UG
p_97	complex_DNA_1nt_5dRP_APE_polBETA_PCNA
p_98	complex_DNA_flapStructure_APE_polBETA
p_99	complex_DNA_1ntgap_FEN1_polBETA
p_100	complex_DNA_unligated_APE_polBETA_FEN1
p_101	complex_DNAligated_polBETA_FEN1_LIG1
p_102	complex_DNAunligated_polBETA_FEN1
p_103	g2_UA_UssDNA
p_104	g3_5hmU_G
p_105	g5_5OHU_ssDNA
p_106	g6_other5OHU
p_107	complex_DNA_NEIL2_8oxoA_TG
p_108	gr7_TU_CpG
p_109	g10_8oxoA_TG
p_110	g11_T_TCG_O6_5fC_5caC
p_111	g8_8oxoA_C
p_112	g12_FaPyG_C
p_113	g18_8oxoG_with_C_T_G
p_114	g13_A_8_8oxoG_C_hydroxA
p_115	g14_137_MeAGC_ethenoA_m6A_hypoxanth
p_116	gr9_5OHC_dsDNA
p_117	g16_thymineGlycol_ex_TgG
p_118	gr23_cytosineGlycol_dsDNA_5hydroxy6hydroT
p_119	g19_5OHC_ssDNA
p_120	gr20_Tg_in_ssDNA
p_121	gr21_Tg_in_dsDNA
p_122	gr22_FaPyA_FaPyG_other
p_123	complexDNA_hSMUG1_g3_5hmU_G
p_124	complexDNA_TDG_5methUrac
p_125	complexDNA_hSMUG1_5hydroxyU
p_126	complexDNA_hSMUG1_otherUrac
p_127	complexDNA_TDG_8oxoA_TG
p_128	_8oxoA_TG
p_129	complex_DNA_NEIL3_5OHC_ssDNA
p_130	complex_DNA_8oxoG_CTG
p_131	complex_DNA_NEIL2_5OHC_dsDNA
p_132	complex_DNA_NEIL2_5OHC_ssDNA
p_133	complex_DNA_NEIL1_thymGlyc_dsDNA
p_134	complex_DNA_NEIL1_5OHC_ssDNA

p_135	complex_DNA_NEIL1_5OHC_dsDNA
p_136	complex_DNA_NEIL1_thymGlyc_ssDNA
p_137	complex_DNA_OGG1_T_TCG_O6_5fC_5caC
p_138	_8oxoG
p_139	_FaPyA_FaPyG_other
p_140	intact_DNA_low_level
p_141	repaired_DNA_after_damage
p_142	DNA_high_level
p_143	gr4_5hmU_ssDNA_5fU
p_144	complexDNA_NEIL3_Tg_in_ssDNA
p_145	NEIL2
p_146	complexDNA_3dRP_NEIL1
p_147	complex_DNA_NEIL1_8oxoAC
p_148	complexDNA_hNTH1_FapyGC
p_149	FaPyG
p_150	complexDNA_TDG_thymineGlycol
p_151	thymineGlycol
p_152	complexDNA_MBD4_thymineGlycol
p_153	complexDNA_hNTH1_thymGlyc_dsDNA
p_154	g17_FapyG_with_A_G_T
p_155	complex_DNA_OGG1_FapyG
p_156	_5_OHC_dsDNA
p_157	Cg_dsDNA_waste
p_158	gr13_waste
p_159	complexDNA_MBD4_gr7
p_160	gr7_waste
p_161	complexDNA_MBD4_5hmU_G
p_162	coplex_DNA_NEIL2_8oxoAC
p_163	_T_TCG_O6_5fC_5caC
p_164	gr15_5OHU_dsDNA_56diHT_56_diOHU
p_165	complexDNA_hNTH1_5OHU_dsDNA_56diHT_56_diOHU
p_166	_5OHU_dsDNA_56diHT_56_diOHU
p_167	complex_DNA_NEIL1_5OHU_dsDNA_56diHT_56_diOHU
p_168	complex_DNA_NEIL2_5OHU_dsDNA_56diHT_56_diOHU
p_169	gr24_FaPyA
p_170	gr25_DNApsoralen
p_171	gr26_55diHU_8oxoG_ssDNA
p_172	FaPyA
p_173	DNApsoralen
p_174	_55diHU_8oxoG_ssDNA
p_175	complex_DNA_NEIL1_FaPyA
p_176	complex_DNA_NEIL2_FaPyA
p_177	complex_DNA_NEIL1_DNApsoralen
p_178	complex_DNA_NEIL2_55diHU_8oxoG_ssDNA

Table B. Biological meaning of all Petri net transitions.

Transition	Biological meaning
t_0	DNA_synthesis_LP_delta
t_1	DNA_synthesis_LP_epsilon
t_2	MBD4_displacement_by_APE
t_3	MPG_displacement_by_APE
t_4	MYH_displacement_by_APE
t_5	NEIL1_liase_activity
t_6	NEIL2_liase_activity
t_7	NEIL3_displacement_byAPE
t_8	NEIL3_displacement
t_9	NEIL3_liase_activity
t_10	OGG1_liase_activity
t_11	OGG1_displacement_by_APE
t_12	PCNA_freed
t_13	PCNA_decreasing
t_14	PCNA_increasing
t_15	TDG_displacement_by_APE
t_16	UNG2_displacement_by_APE
t_17	cleavage_gr14_by_MPG
t_18	cleavage_5hydroxyU_by_NEIL1
t_19	cleavage_5hydroxyU_by_NEIL2
t_20	cleavage_5hydroxyU_by_NEIL3
t_21	cleavage_5hydrouracil_by_UNG2
t_22	cleavage_gr13_by_MYH
t_23	cleavage_8oxyG_by_NEIL1
t_24	cleavage_FapyG_by_hNTH1
t_25	cleavage_FapyGC_by_NEIL1
t_26	cleavage_FaPyA_FaPyG_other_by_NEIL3
t_27	cleavage_FoPy_by_OGG1
t_28	cleavage_8oxoG_by_OGG1
t_29	cleavage_Cg_dsDNA_by_hNTH1
t_30	cleavage_5OHC_dsDNA_by_hNTH1
t_31	cleavage_5OHC_dsDNA_by_TDG
t_32	cleavage_of_APs_by_APE
t_33	cleavage_thymGlic_by_NEIL1
t_34	cleavage_thymGlyc_by_hNTH1
t_35	cleavage_8oxoAC_by_TDG
t_36	cleavage_5hmU_ssDNA_by_MBD4
t_37	cleavage_uracil_by_TDG
t_38	cleavage_uracil_by_UNG2
t_39	cleavage_uracil_by_hSMUG1
t_40	disposal_of_PPi
t_41	incorporation_1nt
t_42	polBeta_incorporates_1nt

t_43	disposal_of_dRP
t_44	disposal_of_dNTP
t_45	hNTH1_displacement_by_APE
t_46	dissociation_SP_complex
t_47	dissociation_LPDelta_complex
t_48	dissociation_LPEpsilon_complex
t_49	flap_cleavage_LP_delta
t_50	flap_cleavage_LP_epsilon
t_51	generate_dNTP
t_52	hNTH1_displacement
t_53	hNTH1_liase_activity
t_54	hSMUG1_displacement_by_APE
t_55	recognMBD4_gr7
t_56	ligase_recruitment
t_57	ligation_LIG3
t_58	ligation_in_LP_delta
t_59	ligation_in_LP_epsilon
t_60	pBETA_displacement_by_pDELTA
t_61	pBETA_displacement_polEPISOLON
t_62	polBETA_recruitment
t_63	recognMPG_of_gr14
t_64	recognMYH_of_gr13
t_65	recognNEIL1_FaPyGC
t_66	recognNEIL1_of_5hydroxyU_ssDNA
t_67	recognNEIL3_of_5hydroxyU_ssDNA
t_68	recognNEIL1_of_8oxyG
t_69	recognNEIL2_of_5hydroxyU_ssDNA
t_70	recognNEIL3_FaPyA_FaPyG_other
t_71	recognNTH1_of_Cg_dsDNA
t_72	recognNTH1_5OHC_dsDNA
t_73	recognNTH1_of_FapyG
t_74	recognNTH1_of_thymineGlycol
t_75	recognTDG_5OHC_dsDNA
t_76	recognTDG_of_8oxoAC
t_77	recognOGG1_of_8oxoG_dmg
t_78	recognOGG1_of_FaPyGC_dmg
t_79	recognTDG_uracilGroup1
t_80	recognUNG2_of_5hydroUra
t_81	recognUNG2_of_OtherUracilGroup2
t_82	recogn_hSMUG1_uracilGroup1
t_83	recognitionNEIL1_of_thymineGlicol
t_84	OGG1_displacement
t_85	removal_3dRP_by_APE
t_86	NEIL1_displacement
t_87	NEIL2_displacement
t_88	APE_decreasing

t_89	APE_increasing
t_90	removal_3P_by_PNKP
t_91	flap_out
t_92	polBeta_recruitment
t_93	polBETA_recruitment_and_1nt_incorp_with_PNKP
t_94	ligation_forPNKP
t_95	dissociation_SP_complex_PKNP_path
t_96	polBETA_removes_5dRP
t_97	disposal_of_3P
t_98	polBeta_incorporates_1nt_PKNP_path
t_99	NEIL1_stimulated_displacement_of_OGG1
t_100	FEN1_dRP_cleavage
t_101	UA_UssDNA_dmgCreation
t_102	PCNA_added_to_the_complex
t_103	DNA_synthesis
t_104	U_UG_dmgCreation
t_105	flap_excision
t_106	polBETA_gap_filling
t_107	APE_removal
t_108	ligation_LIG1
t_109	dissociation_otherLP
t_110	_5hmU_G_dmgCreation
t_111	_5OHU_ssDNA_dmgCreation
t_112	other_5OHU_dmgCreation
t_113	recognNEIL2_of_8oxoA_TG
t_114	TU_CpG_dmgCreation
t_115	_8oxoA_TG_dmgCreation
t_116	_8oxoA_C_dmgCreation
t_117	FaPyGC_dmgCreation
t_118	_8oxoG_with_CGT_dmgCreation
t_119	T_TCG_O6_5fC_5caC
t_120	A_8_8oxoC_C_hydroxA_dmgCreation
t_121	_137_MeAGC_ethenoA_m6A_hypoxanth_dmgCreation
t_122	_5OHC_dsDNA
t_123	thymineGlycol_ex_TgG_dmgCreation
t_124	FapyG_with_A_G_T_dmgCreation
t_125	cytosineGlycol_dsDNA__5hydroxy6hydroT_dmgCreation
t_126	_5OHC_ssDNA_dmgCreation
t_127	Tg_in_ssDNA_dmgCreation
t_128	Tg_in_dsDNA_dmgCreation
t_129	NEIL3_others_damages_dmgCreation
t_130	recogn_hSMUG1_uracilGroup2
t_131	recogn_hSMUG1_g3_5hmUG_gr3
t_132	cleavege_g3_5hmUG_by_hSMUG1
t_133	recognTDG_of_5methUrac_gr3
t_134	cleavage_5methUrac_by_TDG

t_135	disposal_of_uracil
t_136	disposal_of_thymineGlycol
t_137	disposal_of_5hydroxyC
t_138	disposal_of_FaPyGC
t_139	recognNEIL3_Tg_in_dsDNA
t_140	disposal_of_8oxoAC
t_141	disposal_of_grp14waste
t_142	recogn_SMUG1_of_5hydroxyU_ssDNA
t_143	cleavage_5hydroxyU_by_hSMUG1
t_144	recogn_hSMUG1_otherUracil_gr6
t_145	cleavage_otherUrac_by_hSMUG1
t_146	recognTDG_8oxoA_TG
t_147	cleavage_8oxoA_TG_by_TDG
t_148	disposal_of_8oxoA_TG
t_149	recognNEIL3_5OHC_ssDNA
t_150	cleavage_5OHC_ssDNA_by_NEIL3
t_151	recognOGG1_of_8oxoG_CTG
t_152	cleavage_8oxoG_CTG_by_OGG1
t_153	recognNEIL2_of_5OHC_ssDNA
t_154	recognNEIL2_5OHC_dsDNA
t_155	cleavage_5OHC_dsDNA_by_NEIL2
t_156	cleavage_5OHC_ssDNA_by_NEIL2
t_157	recognNEIL1_thymGlyc_dsDNA
t_158	recognNEIL1_5OHC_ssDNA
t_159	cleavage_thymGlyc_dsDNA_by_NEIL1
t_160	cleavage_5OHC_ssDNA_by_NEIL1
t_161	recognNEIL1_of_5OHC_dsDNA
t_162	recognNEIL1_thymGlyc_ssDNA
t_163	cleavage_5OHC_dsDNA_by_NEIL1
t_164	cleavage_thymGlyc_ssDNA_by_NEIL1
t_165	disposal_of_8oxoG
t_166	recognTDG_T_TCG_O6_5fC_5caC
t_167	cleavage_T_TCG_O6_5fC_5caC_by_OGG1
t_168	disposal_of_FaPyA_FaPyG_other
t_169	DNA_back_to_pool
t_170	intact_DNA_increasing
t_171	intact_DNA_decreasing
t_172	_5hmu_ssDNA_5fU_dmgCreation
t_173	recogn_hSMUG1_5hmU_ssDNA
t_174	recogn_MBD4_5hmU_ssDNA_gr4
t_175	recognNEIL3_Tg_in_ssDNA
t_176	cleavage_Tg_in_ssDNA_by_NEIL3
t_177	NEIL1_liase_activity_in_3dRP
t_178	APE_in
t_179	APE_out
t_180	polBETA_in

t_181	polBETA_out
t_182	polDELTA_in
t_183	polDELTA_out
t_184	polEPSILON_in
t_185	polEPSILON_out
t_186	LIG1_in
t_187	LIG1_out
t_188	LIG3_XRCC1_in
t_189	LIG3_XRCC1_out
t_190	OGG1_in
t_191	OGG1_out
t_192	NEIL1_in
t_193	NEIL1_out
t_194	NEIL2_in
t_195	NEIL2_out
t_196	NEIL3_in
t_197	NEIL3_out
t_198	hNTH1_in
t_199	hNTH1_out
t_200	MPG_in
t_201	MPG_out
t_202	UNG2_in
t_203	UNG2_out
t_204	MBD4_in
t_205	MBD4_out
t_206	TDG_in
t_207	TDG_out
t_208	MYH_in
t_209	MYH_out
t_210	hSMUG1_in
t_211	hSMUG1_out
t_212	recognNEIL1_of_8oxoAC
t_213	cleavage_8oxoAC_by_NEIL1
t_214	disposal_of_FapyG
t_215	recognNTH1_of_FaPyGC
t_216	cleavage_FapyGC_by_hNTH1
t_217	recognTDG_thymineGlycol
t_218	cleavage_thymineGlycol_by_TDG
t_219	cleavage_thymineGlycol_by_MBD4
t_220	recognMBD4_thymineGlycol
t_221	recognNTH1_thymGlyc_dsDNA
t_222	cleavage_thymGlyc_dsDNA
t_223	recognOGG1_FapyG
t_224	cleavage_FapyG_by_OGG1
t_225	disposal_of_5OHC_dsDNA
t_226	disposal_of_gr23waste

t_227	disposal_of_gr13waste
t_228	cleavage_gr7_by_MBD4
t_229	disposal_of_gr7waste
t_230	recogn_MDB4_g3_5hmUG_gr3
t_231	recognNEIL2_8oxoAC
t_232	cleavage_8oxoAC_by_NEIL2
t_233	cleavage_8oxoA_TG_by_NEIL2
t_234	disposal_of_T_TCG_O6_5fC_5caC
t_235	_5OHU_dsDNA_56diHT_56_diOHU_dmgCreation
t_236	recognNTH1_5OHU_dsDNA_56diHT_56_diOHU
t_237	cleavage_5OHU_dsDNA_56diHT_56_diOHU_by_hNTH1
t_238	disposal_of_5OHU_dsDNA_56diHT_56_diOHU
t_239	recognNEIL1_of_5OHU_dsDNA_56diHT_56_diOHU
t_240	cleavage_5OHU_dsDNA_56diHT_56_diOHU_by_NEIL1
t_241	recognNEIL2_of_5OHU_dsDNA_56diHT_56_diOHU
t_242	cleavage_5OHU_dsDNA_56diHT_56_diOHU_by_NEIL2
t_243	cleavage_Tg_in_dsDNA_by_NEIL3
t_244	FaPyA_dmgCreation
t_245	DNApsoralen_dmgCreation
t_246	_55diHU_8oxoG_ssDNA_dmgCreation
t_247	disposal_of_FaPyA
t_248	disposal_of_DNApsoralen
t_249	disposal_of_55diHU_8oxoG_ssDNA
t_250	recognNEIL1_FaPyA
t_251	cleavage_of_FaPyA_by_NEIL1
t_252	recognNEIL2_FaPyA
t_253	cleavage_of_FaPyA_by_NEIL2
t_254	recognNEIL1_of_DNApsoralen
t_255	cleavage_DNApsoralen_by_NEIL1
t_256	recognNEIL2_of_55diHU_8oxoG_ssDNA
t_257	cleavage_55diHU_8oxoG_ssDNA_by_NEIL2
t_258	cleavage_5hmU_G_by_MBD4
