# Supplementary material for: Petri net–based model of the human DNA base excision repair pathway
Source: PLoS One. 2019 Sep 13;14(9):e0217913. doi: 10.1371/journal.pone.0217913 (PMC6743755; doi:10.1371/journal.pone.0217913)
Supplement: S1 Appendix — (PDF) [file pone.0217913.s002.pdf]

Appendix 1

Petri nets

Petri net is a mathematical object, whose structure is a directed bipartite graph. The set of vertices of such a graph can be divided into two disjoint subsets in such a way that every arc of the graph is incident with vertices being elements of both of these subsets (i.e., there is no arc being a pair of vertices from the same subset). In Petri nets vertices belonging to one of these subsets are called places while the vertices belonging to the second subset are called transitions. When a Petri net is a model of some system, places correspond to passive system components, as chemical compounds, while transitions correspond to active system components, as chemical reactions or other elementary subprocesses. Places can hold objects called tokens, which represent discrete quantities of the passive components. The arcs are labeled by positive integer numbers called weights. In graphical representation of Petri nets places are denoted as circles, transitions as rectangles or bars, arcs as arrows and tokens as dots or positive integer numbers located in places. If a weight of an arc in equal to one, usually it is not shown in the graphical representations of the net, otherwise it is represented as a number labeling the arc.

The bipartite graph underlying a Petri net determines its structure but most of the fundamental properties of nets of this type follows from their dynamics. This dynamic is related to tokens. Tokens are objects residing in places and flowing through the net from one place to another *via* transitions what corresponds to the flow of information, substances, etc. through the modeled system. The number of tokens residing in a place may vary and the distributions of tokens over all places of the net corresponds to a state of the modeled system. This distribution is called a marking.

The flow of tokens is governed by a simple transition firing rule, according to which a transition is enabled if in all its pre-places, i.e., those ones which directly precede this transition, the number of tokens is equal to at least the weight of an arc connecting this place with the transition. An enabled transition may fire, what means that tokens flow from its pre-places to its post-places, i.e., those ones which are its immediate successors. The number of tokens flowing between two vertices of the net is equal to the weight of an arc connecting them.

There are two exceptions to this rule. One of them concerns transitions without pre-places. They are called input transitions and are continuously enabled. The second one concerns transitions without post-places, called output transitions. Transitions of this type do not produce any tokens when fired. Transitions of both of these types can be used to model interactions of the system with its environment.

Although the graphical representation of Petri nets is intuitive and helpful at the stages of the development and simulation of the net, it is not a good basis for the formal analysis of its properties. For this purpose another representation, called incidence matrix $A = [a_{i,j}]_{n \times m}$ is used. The rows of this matrix correspond to places while columns correspond to transitions. Entries of matrix A are integer numbers and entry $a_{i,j}$ is equal to the difference between numbers of tokens in place p_i before and after firing transition t_j.

There is a lot of Petri nets properties important for their behavior and the behavior of the modeled system, however, for the analysis of Petri net based models of biological systems the especially important are properties related to invariants of the net. There are two types of such invariants, i.e., t-invariants and p-invariants. An invariant of the former type is a solution to the equation

$$A \cdot x = 0$$

while a p-invariant is a solution to the equation

$$A^{T} \cdot y = 0.$$

From this follows that every invariant is a vector of integers, but it corresponds to some subset of the set of transitions or places. This subset is called a support of an invariant. Formally, a support of t-invariant x is set $supp(x) = \{t_j > 0, j=1,2,\ldots,m\}$. A t-invariant is minimal if its support does not properly contain a support of another t-invariant. A support of p-invariant and a minimal p-invariant are defined analogously. Minimal t- and p-invariants are quite important because every other t- or p-invariant can be obtained as a linear combination of the minimal ones. So, in many cases it is sufficient to consider only the minimal invariants.

A support of a t-invariant is a set of transitions whose firing a proper number of times (i.e., a number of times equal to an entry in the invariant corresponding to a given transition) does not change the marking of the net (i.e., it does not change the state of the modeled system).

A support of a p-invariant is a set of places whose weighted number of tokens does not change (a weight is equal to an entry in the p-invariant corresponding to a given place).

A Petri net which is a model of some biological system should be covered by t-invariants what means that every transition should belong to a support of at least one t-invariant. In such a case, every transition influences a behavior of the net, hence every modeled elementary subprocess contribute to a behavior of the system.

t-invariants correspond to some subprocesses of the modeled biological systems. Hence, analyzing relations among them may lead to discoveries of some interactions between the corresponding biological subprocesses.

These relations follow from the fact that supports can have non-empty intersections. Transitions being elements of the intersections correspond to some elementary subprocesses common to subprocesses related to the supports. It means that some subprocesses may influence each other by such a common elementary subprocesses. Hence, finding similar t-invariants (i.e., those whose supports have relatively large intersections) may lead to interesting conclusions concerning interactions and dependencies present in the analyzed biological system. Obviously, in order to do such an analysis every t-invariant should have assigned its biological interpretation.

In order to find the above-mentioned relations t-invariants are grouped into sets called t-clusters. Such a grouping can be done using one of the standard clustering algorithms. The resulting clusters correspond to some functional modules of the analyzed system whose biological meaning should also be determined.

Also transitions can be grouped into sets called Maximal Common Transition sets (MCT sets for short) what helps the analysis of t-invariants. An MCT set is composed of transitions belonging to exactly the same t-invariants. Sets of this type partition the set of transitions into disjoint subsets which correspond to some functional modules of the system (similarly like t-clusters), therefore a biological meaning of such subsets can be described. Since every MCT set is composed of transitions, t-invariants can be seen as collections of MCT sets and single transitions.
